# Molecular Mapping to Species Level of the Tonsillar Crypt Microbiota Associated with Health and Recurrent Tonsillitis

**DOI:** 10.1371/journal.pone.0056418

**Published:** 2013-02-21

**Authors:** Anders Jensen, Helena Fagö-Olsen, Christian Hjort Sørensen, Mogens Kilian

**Affiliations:** 1 Department of Biomedicine, Faculty of Health Sciences, Aarhus University, Aarhus, Denmark; 2 Department of Oto-Rhino-Laryngology, Head and Neck Surgery, Copenhagen University Hospital Gentofte, Copenhagen, Denmark; Virginia Commonwealth University, United States of America

## Abstract

The human palatine tonsils, which belong to the central antigen handling sites of the mucosal immune system, are frequently affected by acute and recurrent infections. This study compared the microbiota of the tonsillar crypts in children and adults affected by recurrent tonsillitis with that of healthy adults and children with tonsillar hyperplasia. An in-depth 16S rRNA gene based pyrosequencing approach combined with a novel strategy that included phylogenetic analysis and detection of species-specific sequence signatures enabled identification of the major part of the microbiota to species level. A complex microbiota consisting of between 42 and 110 taxa was demonstrated in both children and adults. This included a core microbiome of 12 abundant genera found in all samples regardless of age and health status. Yet, *Haemophilus influenzae*, *Neisseria* species, and *Streptococcus pneumoniae* were almost exclusively detected in children. In contrast, *Streptococcus pseudopneumoniae* was present in all samples. Obligate anaerobes like *Porphyromonas*, *Prevotella*, and *Fusobacterium* were abundantly present in children, but the species diversity of *Porphyromonas* and *Prevotella* was larger in adults and included species that are considered putative pathogens in periodontal diseases, i.e. *Porphyromonas gingivalis*, *Porphyromonas endodontalis*, and *Tannerella forsythia.* Unifrac analysis showed that recurrent tonsillitis is associated with a shift in the microbiota of the tonsillar crypts. *Fusobacterium necrophorum, Streptococcus intermedius* and *Prevotella melaninogenica*/*histicola* were associated with recurrent tonsillitis in adults, whereas species traditionally associated with acute tonsillitis like pyogenic streptococci and *Staphylococcus aureus* were scarce. The findings suggest that recurrent tonsillitis is a polymicrobial infection in which interactions within consortia of taxa play an etiologic role. The study contributes to the human microbiome data, to the understanding of the etiology of infections affecting the tonsils, and forms a basis for further insight into the consequences of the intense microbe-host interactions that take place in the tonsils.

## Introduction

Recent evidence supports the hypothesis that the human body and the commensal microbiota of the skin and mucosal membranes constitute an integrated superorganism [1]. The mutual benefits for the host and microbes are numerous. While the microbes are provided with a selective environment flooded with nutrients, they, in return, play a role in providing a barrier against potential pathogens, in regulation of the epithelial angiogenesis and homeostasis, and in activating and shaping the mucosal and systemic immune systems and their reaction patterns. As a consequence, the composition of the commensal microbiota conceivably plays a role in determining the individual’s disposition for infectious, and autoimmune and allergic diseases [2]–[4].

Understanding of the interactions within the human superorganism and their consequences for health and disease requires detailed information on the composition of the microbiota of the many distinct ecosystems that make up the human body. Among these, the paired palatine tonsils of the Waldeyer’s ring are of particular interest. Due to their location, the palatine tonsils and their lymphoid follicles with M cells similar to those of the Peyer’s patches of the gut, located within the deeply branched crypts, are among the first handling sites for microbial and environmental antigens in the human body. In addition, they are the focus and probable entry site for numerous infections particularly during childhood and adolescence [5], [6].

Tonsillitis is a very common infection of the palatine tonsils mostly in children and young adults and especially recurrent tonsillitis is a clinical problem and many patients may ultimately undergo tonsillectomy. *Streptococcus pyogenes* and other *Streptococcus* species like *S. dysgalactiae* subsp. *equisimilis* have received particular attention as etiological agents [7], [8], but recently, the anaerobic species *Fusobacterium necrophorum* was shown to be associated with tonsillitis and especially recurrent tonsillitis in young adults [9], [10]. While a single microbial species may cause acute tonsillitis, it has been suggested that recurrent tonsillitis is a consequence of a polymicrobial infection with anaerobic bacteria forming a biofilm in the crypts [11], [12]. Although many culture-based studies have compared the bacterial community in the tonsillar crypts from patients with recurrent tonsillitis and patients with hypertrophy [13]–[16], no comprehensive studies using high-resolution molecular techniques have been performed. Over the last few years, DNA pyrosequencing has become widely available and now outperforms other molecular strategies with a sampling depth that is several magnitudes higher [17]. It is now the technique of choice for 16S rRNA bacterial diversity analysis and has been used with success to determine the bacterial diversity within many human compartments including the gut and the oral cavity in relation to health status [18]–[21].

In this study we compared the bacterial diversity within the crypts of the palatine tonsils of children and adults with recurrent tonsillitis and healthy adults and children with tonsillar hyperplasia by using a high-throughput multiplexed 16S rRNA gene pyrosequencing approach. Using a novel strategy we were able to identify the major part of the microbiota to species level. Apart from describing bacterial communities associated with recurrent tonsillitis, the study provides an improved foundation for understanding the significance of the microbiota of the tonsils in health and disease.

## Results

An overview of the 20 subjects, their gender, age, and clinical status, and the number and characteristics of the sequences generated from each sample are shown in Table 1. The number of raw sequences for each sample ranged between 15,461 for sample 2 to 163,955 sequences for sample 16 and had a mean length of 517 bases. Quality filtering excluded in average 43% of the sequences and the number of sequences used for further analyses ranged from 4,599 sequences in sample 20 to 94,893 in sample 16 and amounted to a total of 890,708 sequences with a mean length of 334 bases. All samples, however, were rarefied to 4599 sequences prior the OTU based analysis.

**Table 1:** Subject characteristics, sequence data and richness and diversity estimate calculations for each sample.

Sample	Group	Gender	Age	Disorder	Raw sequences	Sequences after quality filtering	OTUs (0.03)^a^	Species identified(% of total sequences)	invsimpson^a^	Shannon index^a^
1	Children with tonsillar hyperplasia	Boy	2	Hyperplasia	81,342	54,758	44	50 (91.5)	9.6	2.6
2		Boy	2	Hyperplasia	15,461	7,965	40	31 (87.6)	4.3	2.1
3		Girl	2	Hyperplasia	149,132	88,976	62	56 (81.2)	5.7	2.5
4		Girl	2	Hyperplasia	106,027	68,281	75	68 (94.4)	4.5	2.4
5		Girl	3	Hyperplasia	43,391	26,633	63	54 (89.5)	9.1	2.7
6	Children with recurrent tonsillitis	Girl	3	Recurrent tonsillitis	44,184	24,994	41	34 (86.4)	3.6	2.0
7		Boy	3	Recurrent tonsillitis	57,171	33,918	58	52 (90.4)	5.9	2.4
8		Girl	4	Recurrent tonsillitis	69,539	38,450	51	53 (99.1)	3.6	2.0
9		Boy	2	Recurrent tonsillitis	122,751	85,936	33	46 (99.2)	1.4	0.7
10		Boy	4	Recurrent tonsillitis	36,044	21,197	43	40 (94.9)	4.4	1.9
11	Healthy adults	Female	31	Benign tumor in throat	60,692	32,708	87	74 (91.4)	9.1	3.0
12		Male	19	Septum plastic	101,804	48,560	102	79 (76.4)	15.0	3.3
13		Female	24	Ear surgery	69,045	42,648	37	38 (94.5)	1.4	0.9
14		Female	35	Polyp on vocal cord	104,770	60,589	56	51 (70.0)	12.1	3.0
15		Female	18	Polyp on vocal cord	41,377	23,194	66	57 (90.4)	6.6	2.6
16	Adults with recurrent tonsillitis	Female	32	Recurrent tonsillitis	163,955	94,893	81	77 (92.4)	12.2	3.0
17		Male	20	Recurrent tonsillitis	86,151	54,434	70	62 (99.5)	11.7	2.8
18		Female	18	Recurrent tonsillitis	94,516	55,480	49	51 (94.4)	5.2	2.2
19		Male	23	Recurrent tonsillitis	112,945	21,329	58	53 (89.5)	4.2	2.1
20		Male	26	Recurrent tonsillitis	56,202	4,599	56	43 (79.4)	10.0	2.7

a) Samples were rarefied to 4599 prior to the analysis.

**Figure 1:**
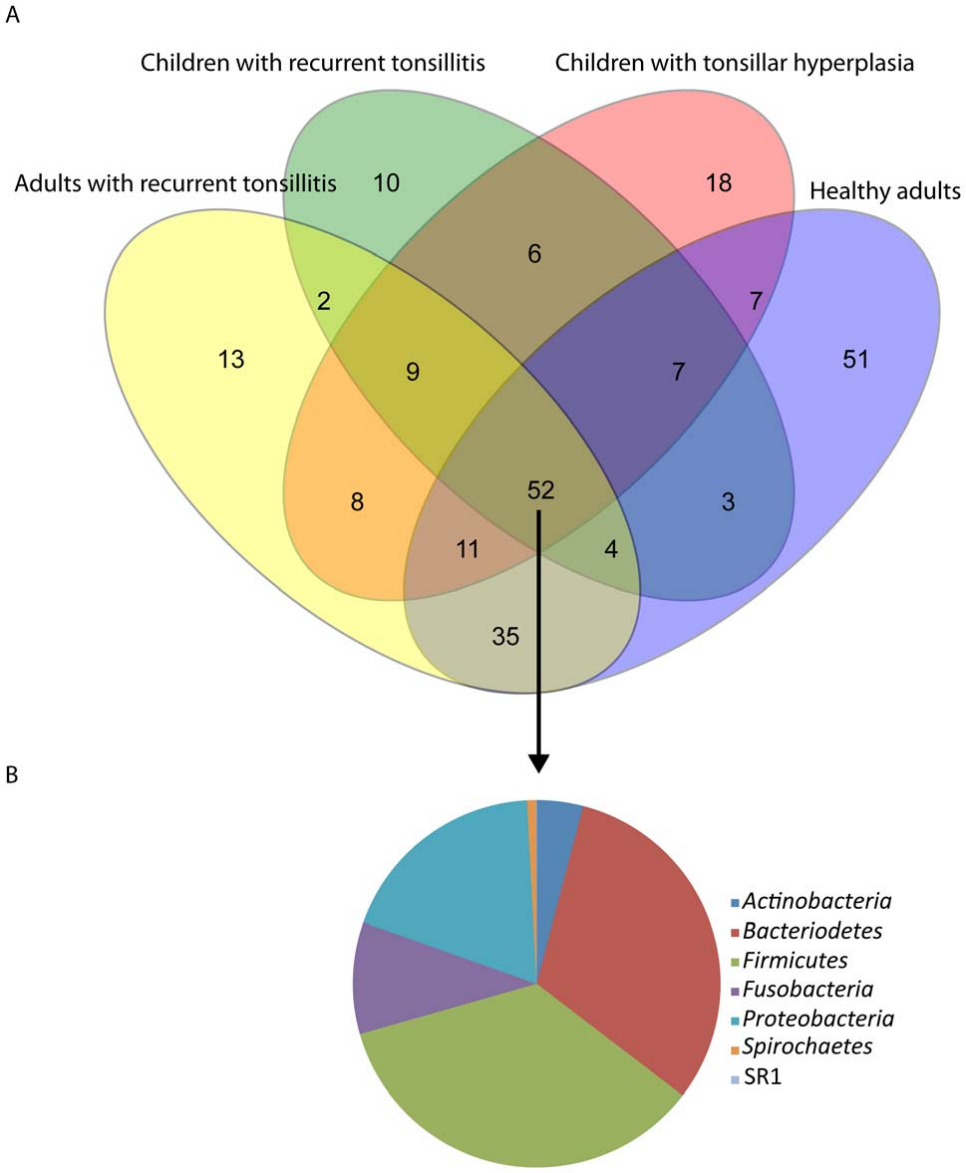
Venn diagram showing overlap between observed OTUs for the four groups. A total of 52 OTUs detected in all four groups accounted for more than 90% of all sequences (A). Data are also expressed according to the phyla, to which the shared OTUs belonged (B).

The number of OTUs at a 3% difference level ranged from 33 in sample 9 to 108 in sample 12 (Table 1). In total, 236 different OTUs were found across all samples, of which 14 OTUs were detected in all 20 samples and accounted for a mean of 64% (range: 6% to 95%) of the total number of sequences. This indicates that a core microbiome is present in the crypts of human tonsils regardless of age and health status of the tonsils. The predominant members of this core microbiota belonged to the genera *Streptococcus*, *Prevotella*, *Fusobacterium, Porphyromonas, Neisseria, Parvimonas, Haemophilus, Actinomyces, Rothia, Granulicatella*, and *Gemella*. When samples were combined into the four groups based on age and clinical characteristics, 52 OTUs accounting for 89% of the total number of sequences where represented in all four groups as illustrated by the Venn diagram (Figure 1). No significant differences (p>0.2) in the community complexity of the four groups and between children and adults were detected by invsimpson and Shannon diversity indexes (Table 1).

**Figure 2:**
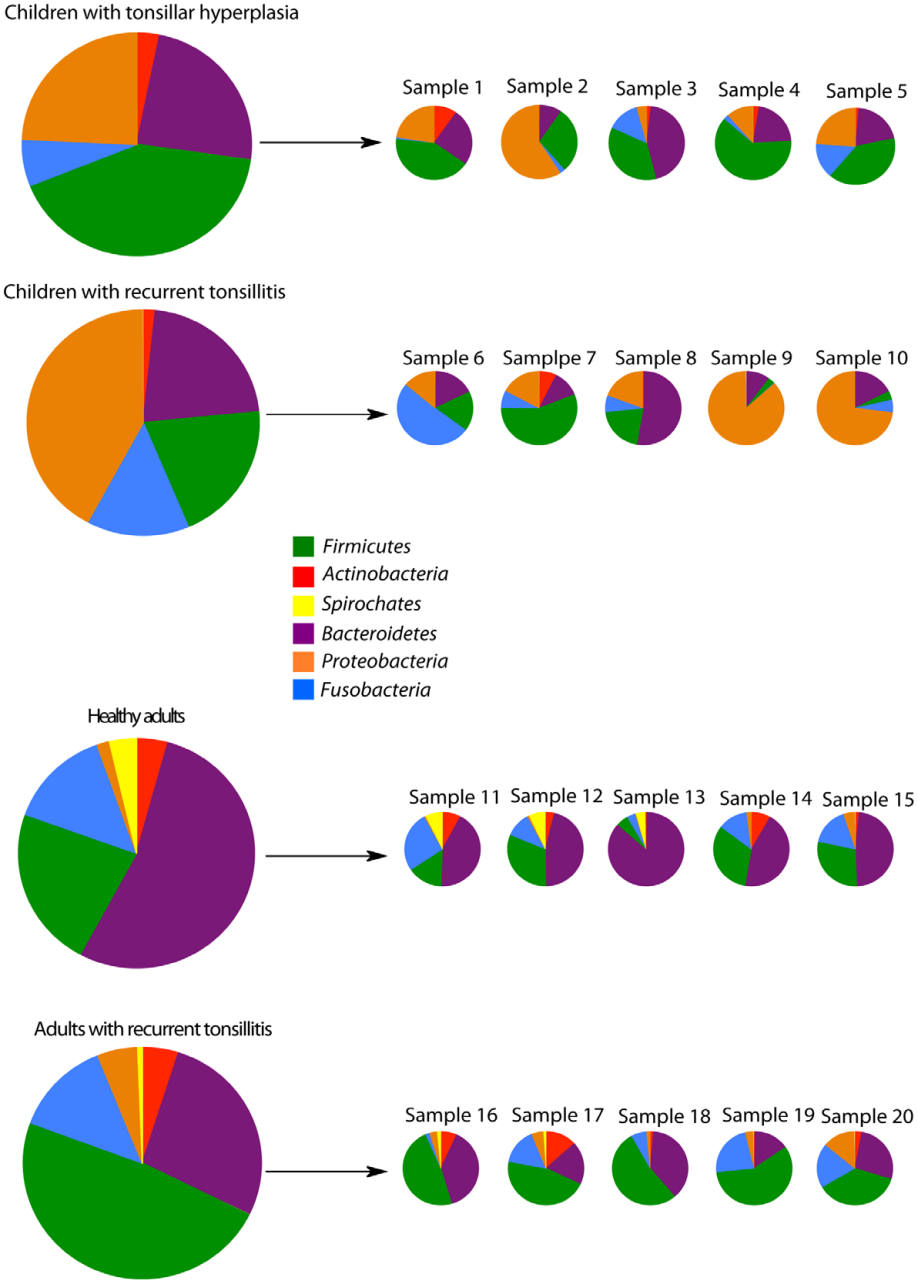
Relative abundance (percentage of total sequences) of the six bacterial phyla found in substantial amounts (>1%) in the four specified groups of adults and children and in the individual samples.

Taxonomic groupings of the sequences showed that ten phyla were detected across all samples (Table S2). However, only *Firmicutes, Bacteroidetes, Proteobacteria, Fusobacteria, Actinobacteria,* and *Spirochaetes* were found in proportions exceeding 1% (Figure 2). Apart from *Spirochaetes,* found only in 13 of the 20 samples, and primarily in healthy adults, the major phyla were found in all samples (Figure 2). Among the minor phyla that were detected, *Cyanobacteria* and *Deinococcus-Thermus* are probably transient contaminants from inhalation and food intake. A minor proportion of the sequences could not be assigned to a known phylum, and may be due to inadequate chimera-detection.

In spite of considerable inter-individual variation (Figure 2), significant differences in the phylum composition between the four groups were detected by metastats. Both *Bacteroidetes* and *Firmicutes* were associated with healthy adults. In contrast, *Proteobacteria* were highly associated with children (Table 2 and table S2).

**Table 2:** Metastats analysis of differential abundance of bacterial taxa detected in tonsillar samples from the four groups of subjects.

Phylum	Genus	Species		
*Proteobacteria*↑^A^	***Aggregatibacter***	***Aggregatibacter segnis***↓^A^		
	*Aquamonas*↓^A^			
	*Eikenella*	*Eikenella corrodens*↓^A^		
	*Enhydrobacter*↑^A^			
	*Escherichia*↑^A^			
	***Haemophilus***↑^A^	***Haemophilus haemolyticus***↑^A^		
		***Haemophilus influenzae***↑^A^		
		***Haemophilus parainfluenzae***↓^B^		
		*Haemophilus paraphrohaemolyticus*↓^A^		
	*Kingella*↑^A^	*Kingella kingae*↑^A^		
		*Kingella potus*↑^A^		
	***Neisseria***↑^A^	***Neiserria cinerea***↑^A^		
		*Neiserria meningitidis*↑^A^		
		*Neiserria mucosa*↑^A^		
*Bacteroidetes*↓^A^	*Odoribacter*↓^A^	*Odoribacter denticanis*↓^A^		
	*Paludibacter*↓^A^	*Paludibacter propionicigenes*↓^A^		
	***Prevotella***↓^A^	*Prevotella bivia*↓^A^		
		*Prevotella buccae*↓^A^		
		*Prevotella dentalis*↓^A^		
		*Prevotella denticola*↓^A^		
		*Prevotella fusca*↓^A^		
		*Prevotella genomospecies* PRAJ6, uncultured↓^A^		
		*Prevotella genomospecies* PRAJ7, uncultured↓^A^		
		*Prevotella genomospecies* PRAJ9, uncultured↓^A^		
		***Prevotella intermedia***↓^A^		
		***Prevotella melaninogenica/P. histicola***↑^C^		
		*Prevotella micans* A		
		***Prevotella nanceiensis***↓^B^		
		*Prevotella oralis*↓^A^		
		***Prevotella oris***↓^A^		
		***Prevotella pallens***↓^A^		
		*Prevotella pleuritidis*↓^A^		
		*Prevotella saccharolytica*↑^A^		
		*Prevotella saliviae*↓^A^		
		***Prevotella veroralis***↓^A^		
	***Porphyromonas***	*Porphyromonas asaccharolytica*↓^A^		
		***Porphyromonas cataniae***↑^A^		
		***Porphyromonas endodontalis***↓^A^		
		***Porphyromonas genomospecies*** ** PAJ1, uncultured**↑^A^		
		***Porphyromonas gingivalis***↓^A^		
	***Tannerella***↓^A^	***Tannerella forsythia***↓^A^↓^C^		
		***Tannerella genomospecies*** ** TAJ1, uncultured**↓^A^		
*Actinobacteria*	***Actinomyces***↓^A^↓^B^	*Actinomyces cardiffensis*↑^A^		
		*Actinomyces massiliensis*↑^A^↓^B^		
		*Actinomyces radicidentis*↑^A^		
	*Adlercreutzia*↓^A^			
	*Alloscardovia*↓^A^			
	*Bifidobacterium*↓^A^			
	*Brevibacterium*↓^A^			
	*Dietzia*↓^A^			
	*Gardnerella*↓^A^			
	*Geodermatophilus*↓^A^			
	*Scardovia*↓^A^			
	*Zimmermanella*↓^A^			
***Firmicutes***↓^B^↑^C^	*Abiotrophia*↑^A^	***Abiotrophia elegans***↑^A^↓^B^		
		*Abiotrophia genomospecies* AAJ1, uncultured↑^A^		
	*Anaerococcus*	*Anaeroglobus geminatus*↓^A^		
	*Centipeda*	*Centipeda periodontii*↓^A^		
	*Dialister*↓^A^	*Dialister invisus*↓^A^		
		*Dialister pneumosintes*↓^A^		
	***Eubacterium***↓^A^			
	*Finegoldia*↑^A^			
	***Gemella***↓^B^↑^C^	*Gemella genomospecies* GAJ1, uncultured↑^C^		
		***Gemella haemolysans/G. morbillorum/G. sanguinis***↓^B^↑^C^		
	***Granulicatella***↑^A^↓^B^			
	*Howardella*↓^A^			
	*Lactobacillus*↓^A^			
	*Lactococcus*↓^A^	*Lactococcus lactis*↓^A^		
	*Megasphaera*↓^A^	*Megasphaera micronuciformis*↓^A^		
	***Mogibacterium***↓^A^			
	*Moryella*↓^A^			
	*Mycoplasma*↓^A^			
	*Oribacterium*↓^A^↓^B^			
	***Parvimonas***↓^A^			
	*Selenomonas*↓^C^	*Selenomonas genomospecies* SAJ1, uncultured↓^A^		
		*Selenomonas sputigena*↓^A^		
	***Solobacterium***↓^A^			
	***Streptococcus***↑^C^	***Streptococcus constellatus***↓^A^		
		*Streptococcus gordonii*↑^B^		
		***Streptococcus intermedius***↑^C^		
		*Streptococcus lactarius/S. peroris*↓^A^		
		*Streptococcus mutans*↑^A^		
		***Streptococcus parasanguinis/S. cristatus/S. australis***↓^B^		
		***Streptococcus pseudopneumoniae***↑^A^		
		***Streptococcus salivarius/S. vestibularis***↓^B^		
	***Veillonella***↓^A^↓^B^	***Veillonella dispar***↓^B^		
		***Veillonella genomospecies*** ** VAJ1, uncultured**↓^B^		
		*Veillonella genomospecies* VAJ2, uncultured↓^A^		
***Fusobacteria***	***Fusobacterium***	*Fusobacterium genomospecies* FAJ1, uncultured↑^A^		
		***Fusobacterium genomospecies*** ** FAJ2, uncultured**↓^B^		
		*Fusobacterium genomospecies* FAJ3, uncultured↓^A^		
		***Fusobacterium necrophorum***↓^A^↑^C^		
		***Fusobacterium nucleatum***↓^C^		
	*Sneathia*↑^A^			
***Spirochaetes***↓^A^↑^C^	***Treponema***↓^A^↑^C^	***Treponema denticola***↓^A^↓^C^		
		*Treponema genomospecies* TRAJ1, uncultured↓^A^		
		*Treponema genomospecies* TRAJ2, uncultured↓^A^		
		*Treponema genomospecies* TRAJ3, uncultured↓^A^↓^C^		
		*Treponema lecithinolyticum*↓^A^		
		***Treponema medium***↓^A^		
		*Treponema socranskii*↓^A^		

A Change in abundance in children compared to adults.

B Change in abundance in children with recurrent tonsilltis compared to children with tonsillar hyperplasia.

C Change in abundance in adults with recurrent tonsilltis compared to healthy adults.

Taxa shown in **bold** indicate an average abundance of more than 0.5% in one of the groups.

At a more detailed taxonomic level, sequences were assigned to 93 named genera (Table S2). A core of 12 genera was present in all samples: *Actinomyces, Rothia, Streptococcus, Gemella, Granulicatella, Johnsonella, Prevotella, Porphyromonas, Fusobacterium Veillonella, Neisseria,* and *Haemophilus*. The most prevalent genera were *Prevotella* (27 named and 9 unnamed species), *Streptococcus* (17 species and non-distinguishable groups of species), *Haemophilus* (6 species), *Fusobacterium* (3 named and 3 unnamed species), *Porphyromonas* (4 named and 2 unnamed species), *Gemella* (4 species and clusters of species), *Neisseria* (7 species and non-distinguishable groups of species), *Veillonella* (3 named species or clusters of species and 2 unnamed species), *Capnocytophaga* (4 species and non-distinguishable groups of species), *Parvimonas* (1 species), *Rothia* (3 species), *Actinomyces* (8 species), and *Treponema* (7 named and 3 unnamed species) (Table S2). Among a total of 3.2% of the sequences that could not be assigned to genus level with a reasonable degree of confidence using the automatic procedure for sequence assignment to genus level 2.4% were successfully assigned to species using the manual phylogenetic approach. The sequences assigned to unclassified *Pasteurellaceae,* unclassified *Neisseriaceae* and unclassified *Flavobacteriaceae* by the automatic procedure were all assignedd to species using this analysis. Sequences identified as unclassified *Bacteroidales* by the automatic approach could not be assigned to recognized genera or species using the manual phylogenetic analysis and were all assigned to uncultured genomogenera or genomospecies based on the level at which the clustering appeared (Table S2). The assignment of occasional sequences to recognized genera by the automatic procedure were not confirmed by the phylogenetic analysis and, accordingly, were reassigned to genomogenera (i.e. *Porphyromonadaceae* genomosgenus POAJ1 and *Flavobacteriaceae* genomogenus FLAJ1) (Table S2).

The tonsillar crypts of children with tonsillar hyperplasia were dominated by the following genera: *Streptococcus* (21.5%), *Neisseria* (13.5%), *Prevotella* (12.0%), *Haemophilus* (10.2%), *Porphyromonas* (9.0%), *Gemella* (8.6%), and *Fusobacterium* species (6.4%) (Table S2). The bacterial communities in the tonsils of adults with recurrent tonsillitis were dominated by the same genera but in significantly altered proportions. Metastats analysis showed that *Haemophilus* and *Neisseria* were significantly more abundant in samples from children than from adults (Table 2). In contrast, *Prevotella, Actinomyces, Parvimonas Veillonella*, and *Treponema* were significantly more abundant in adults compared to children (Table 2).

As recurrent tonsillitis conceivably is caused by a shift in the proportion of bacteria at a more detailed phylogenetic level than genera, we used a novel strategy to assign the predominant clusters of sequences to species level by phylogenetic analysis with inclusion of 16S rRNA gene sequences of relevant designated type strains. As an adjunct to this analysis and when the clustering of sequences with single type strains was inconclusive, species-specific nucleotide signatures were used to assign these sequences to species or, in some cases, clusters of species. Thirty-three species exceeded an average mean abundance of more than 0.5% (Table 3). However, considerable inter-individual variation in the abundance of species was observed, and no species constituted more than 0.5% in all 20 samples. Some species, however, constituted more than 0.5% in all samples belonging to one of the four groups. These included *Haemophilus influenzae* in children, *Fusobacterium nucleatum* in healthy adults, *Gemella haemolysans/G. morbillorum/G. sanguinis* in children with hyperplasia and adults with recurrent tonsillitis, the yet uncultured *Porphyromonas* genomospecies PAJ1 in children with hyperplasia, *P. cataniae* in children, *H. haemolyticus* in children with hyperplasia, *F. necrophorum* in adults with recurrent tonsillitis, and *Abiotrophia elegans* in children with hyperplasia (Table 2). In addition, uncultured genomospecies and genomogenera were found in or associated with many genera including *Prevotella* (9 genomospecies), *Fusobacterium* (three genomospecies), *Porphyromonas* (two genomospecies), *Gemella* (two genomspecies), *Tanerella* (one genomospecies), *Abiotrophia* (one genomospecies), *Capnocytophaga* (one genomospecies), *Selenomonas* (one genomospecies), *Veillonella* (two genomospecies), *Treponema* (three genomospecies), *Porphyromonadaceae* (two genomogenus) *and Flavobacteriaceae* (one genomogenus) (Table S2). Sequence identities detected by Blast analysis showed that some of these genomospecies and genomogenus have been previously detected in other microbiome studies. Their closest matches in the HOMD database are listed in Table S3. However, some differences in sequence assignment between the automatic approach and the manual phylogenetic approach were observed (Table S2). The distribution of the species within major genera is described in detail below.

**Table 3:** Absolute abundance expressed in percentage of the total sequences in each sample of species exceeding an average mean of more than 0.5%.

Bacterial species	Children with hyperplasia					Children with recurrent tonsillitis					Healthy adults					Adults with recurrent tonsillitis				
	1	2	3	4	5	6	7	8	9	10	11	12	13	14	15	16	17	18	19	20
**Phylum ** ***Actinobacteria***																				
*Actinomyces odontolyticus*	1.418	0.209	0.462	0.652	0.045	0.080	0.530	0.086	0.019	0.000	0.342	0.764	0.035	4.512	0.470	1.877	3.243	0.991	0.056	0.348
*Rothia mucilaginosus*	4.917	0.093	0.668	1.046	0.248	0.308	0.578	0.195	0.021	0.055	0.135	0.167	0.038	0.403	1.017	4.177	9.286	0.272	0.047	0.239
**Phylum ** ***Bacteroidetes***																				
*Capnocytophaga leadbetteri*	0.000	0.012	0.002	0.012	1.337	0.000	0.684	0.034	0.000	0.549	0.000	0.097	0.000	0.000	0.013	0.005	0.000	29.856	0.801	0.608
*Porphyromonas cataniae*	12.362	4.216	0.738	10.160	0.534	12.676	0.605	0.888	5.114	1.888	0.012	0.176	0.000	0.925	0.826	0.030	0.000	1.329	0.131	3.481
*Porphyromonas endodontalis*	0.000	0.000	0.000	0.000	0.000	0.000	0.000	0.000	0.000	0.000	0.095	11.745	0.000	0.000	32.189	2.928	0.447	0.000	0.000	0.021
*Prevotella intermedia*	0.000	0.000	0.000	0.000	0.000	0.000	0.000	0.000	0.000	0.000	0.223	0.111	84.642	0.000	0.194	0.000	0.000	0.000	0.000	0.000
*Prevotella melaninogenica/P. histicola*	2.018	0.185	38.750	2.733	0.849	0.328	0.666	0.424	4.650	13.349	2.373	0.529	0.026	0.418	0.483	5.138	9.928	2.244	0.984	4.541
*Prevotella nanceiensis*	0.677	0.301	0.100	0.706	0.086	0.076	0.003	0.239	0.049	0.097	0.000	0.097	0.021	0.003	0.246	0.118	0.055	0.076	4.958	4.475
*Prevotella nigrescens*	0.000	0.000	0.000	0.000	1.078	0.000	0.000	0.000	0.000	0.000	0.058	15.060	0.000	0.000	0.000	7.629	0.042	0.013	0.000	0.000
*Prevotella oris*	0.000	0.000	0.000	0.004	0.000	0.000	0.000	0.117	0.000	0.328	4.773	0.187	0.002	0.000	0.000	4.153	0.329	0.034	6.078	0.608
*Prevotella tannerae*	0.000	0.000	0.000	0.035	0.000	0.000	0.000	0.000	0.000	0.000	14.755	0.006	0.002	0.000	0.241	0.003	4.104	0.000	0.000	0.000
*Porphyromonas* genomospecies FAJ1, uncultured	1.963	2.954	2.879	0.920	6.498	1.456	7.687	48.444	0.021	0.075	0.018	0.010	0.000	0.281	0.014	0.000	0.000	1.324	0.145	0.000
*Prevotella* genomospecies PRAJ2,uncultured	0.575	0.324	0.006	0.264	3.717	0.000	0.027	0.000	0.014	0.000	0.003	0.000	0.000	16.592	0.000	0.040	0.000	0.000	0.000	0.000
*Prevotella* genomospecies PRAJ3,uncultured	3.251	0.301	0.695	2.232	1.374	1.088	1.202	0.970	0.035	0.092	0.000	0.010	0.000	1.381	0.069	1.921	0.000	0.000	0.347	0.348
*Prevotella veroralis*	0.000	0.000	0.011	0.135	0.150	0.000	0.021	0.010	0.000	0.000	0.648	0.177	0.000	4.400	1.453	8.945	0.000	0.169	0.000	0.000
**Phylum ** ***Firmicutes***																				
*Gemella haemolysans/G. morbillorum/G. sanguinis*	11.944	4.946	2.474	3.909	17.745	2.921	2.428	4.528	0.158	0.268	0.339	0.906	0.462	2.294	0.780	3.508	6.329	4.762	2.245	3.715
*Granulicatella elegans*	3.011	2.722	0.629	1.498	1.457	0.504	0.907	0.718	0.077	0.028	0.027	0.025	0.000	0.003	0.022	0.042	0.000	0.800	0.005	0.043
*Parvimonas micra*	0.000	0.209	4.726	1.741	0.372	0.004	3.032	0.362	0.000	0.222	2.950	0.070	0.267	0.647	10.136	1.625	0.400	0.000	7.058	17.054
*Streptococcus intermedius*	0.009	0.000	0.004	0.485	0.000	0.000	8.146	0.014	0.008	0.042	0.006	0.078	0.000	0.000	0.065	2.546	0.000	6.696	44.100	8.698
*Streptococcus mitis/S. oralis/S. infantis*	0.074	0.063	0.054	1.916	0.113	0.000	1.683	0.066	0.023	0.002	0.862	0.130	0.106	3.573	0.073	0.455	0.039	28.408	0.000	0.391
*Streptococcus parasanguinis/S. cristatus/S. australis*	3.222	0.452	0.602	0.975	0.222	0.160	0.262	0.297	0.107	0.005	0.339	1.254	0.045	1.700	0.569	3.125	7.062	1.357	0.066	0.217
*Streptococcus pneumoniae*	0.015	0.000	0.067	42.971	0.094	0.000	34.754	0.217	0.172	0.000	0.000	0.000	0.000	0.003	0.000	11.691	0.006	0.000	0.000	0.000
*Streptococcus pseudopneumoniae*	12.722	17.238	5.740	0.117	10.930	2.225	0.212	14.290	1.555	0.175	0.003	2.735	0.087	0.015	6.678	2.753	4.007	0.139	0.277	0.065
*Streptococcus salivarius/S. vestibularis*	3.246	0.578	2.214	2.373	0.207	0.612	0.469	0.684	0.601	0.007	0.263	2.570	0.152	4.836	1.923	12.607	11.647	1.384	0.366	2.305
*Veillonella dispar*	1.086	0.000	1.415	2.020	0.991	0.236	0.383	0.164	0.014	0.439	1.978	1.215	0.033	8.193	1.259	3.543	8.208	1.218	0.178	0.369
**Phylum ** ***Fusobacteria***																				
*Fusobacterium necrophorum*	0.000	0.000	0.000	0.004	0.007	0.000	0.000	0.000	0.000	0.000	0.000	0.002	0.000	0.000	0.005	1.061	4.350	3.205	15.166	18.051
*Fusobacterium nucleatum*	0.000	0.000	12.421	1.398	14.155	49.642	7.532	5.840	0.000	4.509	26.493	10.591	3.580	3.885	16.229	0.464	11.505	5.610	0.313	0.153
**Phylum ** ***Proteobacteria***																				
*Haemophilus haemolyticus*	1.274	2.825	0.390	0.559	0.285	0.084	3.134	8.973	0.071	0.302	0.015	0.058	0.000	0.520	0.043	0.008	0.011	0.768	0.066	0.239
*Haemophilus influenzae*	19.057	2.888	0.804	2.611	18.597	0.588	9.166	7.415	85.366	40.643	0.101	0.000	0.000	0.796	0.121	0.272	0.000	0.124	1.528	0.000
*Haemophilus parainfluenzae*	0.517	0.138	0.266	0.410	0.383	0.052	0.059	0.286	0.098	0.080	0.199	0.210	0.082	0.000	0.884	1.193	4.947	0.342	0.192	0.109
*Neiserria cinerea*	0.013	0.000	0.088	0.000	0.009	0.218	3.339	0.580	0.030	11.876	0.000	0.000	0.000	0.000	0.000	0.000	0.000	0.000	0.000	0.000
*Neiserria elongata/Kingella denitrificans*	0.000	0.000	0.000	0.028	0.000	0.000	1.366	0.005	0.004	15.355	0.000	0.003	0.000	0.018	0.000	0.009	0.109	0.000	0.000	0.112
*Neiserria subflava/N. flavescens*	0.681	0.044	2.016	4.033	0.000	11.199	0.038	1.649	0.406	0.877	0.000	1.495	0.006	0.007	0.342	0.366	0.457	0.216	0.408	2.858
*Neisseria lactamica*	0.575	44.309	0.000	2.065	0.000	0.000	0.000	0.000	0.000	0.000	0.012	0.111	0.007	0.005	0.010	0.000	0.022	0.000	0.000	0.000
**Phylum ** ***Spirochaetes***																				
*Treponema medium*	0.000	0.000	0.000	0.000	0.000	0.000	0.027	0.000	0.114	0.000	5.280	2.658	2.239	0.000	0.000	1.171	0.410	0.000	0.183	0.217

Significant variations between samples were observed. See Table S2 for a complete list of phyla, genera and species identified in the study.

The genus *Haemophilus* was dominated by *H. influenzae* and *H. haemolyticus* in all samples from children and no major differences were observed between children with tonsillar hyperplasia and children with recurrent tonsillitis (Table S2). In contrast, the distribution of *Neisseria* species differed, but not statistically significantly, between children with tonsillar hyperplasia and children with recurrent tonsillitis. Samples from children with recurrent tonsillitis were dominated by *N. cineria, N. flavescens*, and *N. elongata*/*Kingella denitrificans* (Table S2).

*S. pneumoniae* (2 samples) and *S. pseudopneumoniae* dominated the genus *Streptococcus* in children. Interestingly, *S. pseudopneumoniae* was generally very abundant in children and was significant more associated with children than adults (q = 0.04) (Table 2). Conversely, the two Anginosus group *Streptococcus* species *S. constellatus* and *S. intermedius* were found mainly in samples from adults. *S. intermedius,* accounted for 2.7–44.1% (mean = 16%) of the total number of sequences in adults with recurrent tonsilltits, which is higher than in healthy adults (q <0.04) (Table 2). Pyogenic streptococci (*S. pyogenes*, *S. agalactiae*, and *S. dysgalactiae* subsp. *equisimilis*) accounted for less than 1% of all sequences assigned to the genus *Streptococcus* in all samples but one (*S. dysgalactiae* subsp. *equisimilis*, 1.3% of all sequences from sample 15) (Table S2).

Phylogenetic analysis of the genus *Fusobacterium* revealed that all samples, except samples from adults with recurrent tonsillitis were dominated by *F. nucleatum* and two uncultured *Fusobacterium* genomospecies designated FAJ1 and FAJ2 (Table S2). In contrast, *F. necrophorum* was found in substantial amounts in all five samples from adults with recurrent tonsillitis, but were virtually absent from all other samples (Table 3). In two of the samples from adult recurrent tonsillitis *F. necrophorum* accounted for more than 15% of all sequences while it accounted for 2–5% of the total number of sequences in the other three samples from these patients. This is significantly different from healthy adults (Table 2).

*Porphyromonas* sequences from children were assigned exclusively to *P. cataniae* and the uncultured *Porphyromonas* genomospecies PAJ1 both of which were significantly associated with children (Table 2). *Porphyromonas* genomospecies PAJ1 formed a distinct and coherent clusterwas found to be closest related most closely related to *P. catoniae* with 95% sequence similarity. No similar sequence was found in HOMD, but “uncultured bacterium clones” in GenBank were 100% identical to a sequence representing *Porphyromonas* genomospecies PAJ1 (Table S3). In adults, species like *P. asaccharolytica* and *P. endodontalis* and the related *Tannerella forsythia* were also found in substantial proportions in samples from adults. *P. gingivalis* was only present in one sample from a healthy adult (Table S2).

While other genera were dominated by relatively few species, *Prevotella* showed considerable species diversity especially in adults. In addition to known species, among which *P. melaninogenica*/*P. histicola* was the most abundant, several genomospecies were found. *P. melaninogenica*/*P. histicola*, which were found in substantial amounts, (Table S2), were significantly associated with recurrent tonsillitis in adults. In addition, several low abundant species differed in occurrence between children and adults (Table 2).

**Figure 3:**
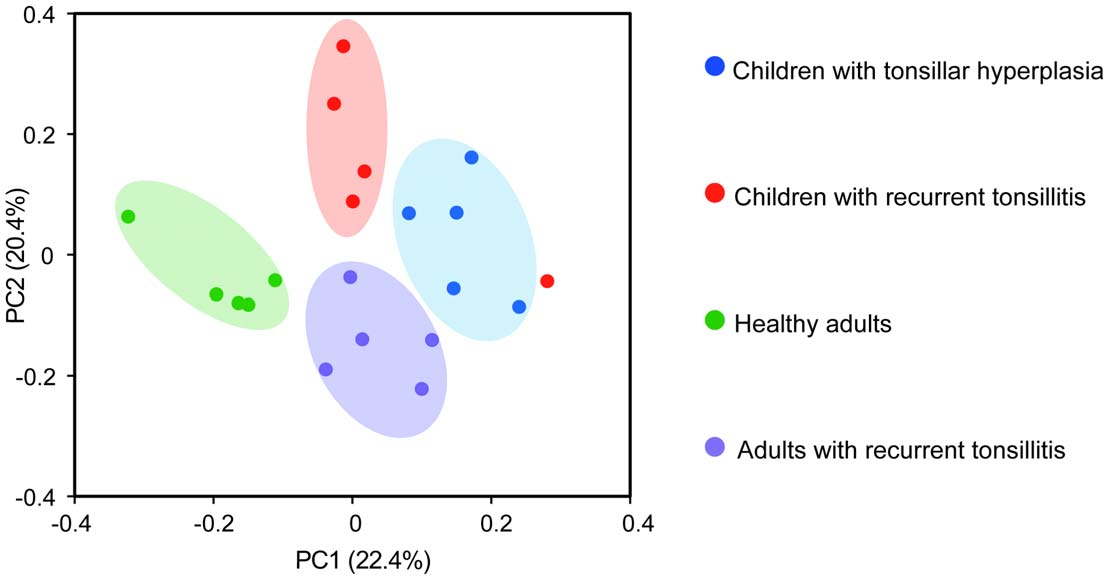
Principal-coordinate analysis (PCoA) plot showing the similarity relations among the 20 tonsillar crypt microbiota compositions. Plots were generated using weighted UniFrac distances using from the phylogenetic tree generated from the Clearcut program. These two components explain 42.8% of the variance. Samples from children with tonsillar hyperplasia are shown as blue circles; children with recurrent tonsillitis as red circles; healthy adults as green circles; and adults with recurrent tonsillitis as purple circles.

To analyse if the phylogenetic community structures were different between the four groups we used UniFrac distances of the phylogenetic tree generated from the Clearcut program. The resulting clustering visualized by a PCoA plot shows that samples clustered according to age and in part the health status (Figure 3). The clustering observed in the PCoA plot was statistically supported by the AMOVA test, which showed that the microbiota of both children with tonsillar hyperplasia and children with recurrent tonsillitis was significantly different from that of healthy adults (p<0.01) and adults with recurrent tonsillitis (p = 0.04; p<0.01, respectively). Furthermore, the AMOVA test revealed a significant difference in the community structure in healthy adults compared to adults with recurrent tonsillitis (p<0.001), whereas no significant difference was observed between children with tonsillar hyperplasia and children with recurrent tonsillitis (p = 0.13). However, when one outlying sample from the children with recurrent tonsillitis group was omitted from the analysis, a significantly different community structure was also evident in these two groups (p = 0.01).

## Discussion

To our knowledge, this is the first report on the complex microbiota of the tonsillar crypts in children and adults assessed by in-depth sequencing. The tonsils are frequent infection foci and the crypt microbiota in both health and disease is in intimate contact with crucial antigen handling lymphoid tissues with yet unknown immunological consequences. While previous studies focused on the surface of the tonsils, we sampled the microbiota of the crypts, made possible by the general anesthesia of the 20 subjects in association with tonsillectomy. One limitation of this procedure is that healthy tonsils from children had to be substituted by hyperplastic tonsils without signs of inflammation and without previous acute or recurrent tonsillitis.

Being aware of the current focus on artefacts that may be introduced by pyrosequencing [27], [28], [34], we applied a stringent protocol for filtering sequences. In addition, the sequences were subjected to phylogenetic analysis combined with detection of species-specific sequence signatures, which, apart from constituting an additional validation step, allowed us to identify a significant part of the microbiota to species level, even species that share more than 97% 16S rRNA gene sequence identity such as several members of the genus *Streptococcus* with distinct clinical significance. Assignment of 16S rRNA sequences to specific taxa is usually done by automated estimation of overall sequence identity with a wide range of reference sequences in databases, some of which are mislabeled [31]. As demonstrated by our results, the detailed taxonomic message of sequences may be missed by that procedure.

Our findings demonstrate that the microbiota of the tonsillar crypts by the age of 2–4 years already has reached the same overall complexity as found in adults as also evidenced by the statistical comparison of the invsimpson and Shannon diversity indexes, yet maintaining striking differences as previously suggested by culture studies [35]. Bacteria that were almost exclusively detected in children were *H. influenzae*, *H. haemolyticus*, *Neisseria* species, and *S. pneumoniae*. Obligate anaerobes like *Porphyromonas*, *Prevotella*, and *Fusobacterium* were abundantly present in children, but the diversity of *Porphyromonas* species was larger in adults and included species that are considered putative pathogens in periodontal diseases [36], i.e. *P. gingivalis*, *P. endodontalis*, and *Tannerella forsythia*.

Only six phyla were present in substantial proportions (>1%) in agreement with findings from other anatomical sites and underlining the selectivity of the colonization of the human body [19], [21], [37]–[39]. One of the major goals of the International Human Microbiome Project is to determine if there is an identifiable ‘core microbiome’ of shared organisms, genes, or functional capabilities in a given body habitat of all or the vast majority of humans [40]. We found that a rather small number of taxa, mostly belonging to the genera *Streptococcus* and *Prevotella,* were present in the tonsillar crypts of all 20 samples and accounted for a substantial proportion of the total number of sequences. Furthermore, the eight most abundant genera accounted for more than 70% of the total number of sequences in all samples. These results shows that a core microbiome is present in the human tonsillar crypts regardless of age and health status and support the general idea of a human core microbiome. Although we included 20 individuals, more individuals have to be analysed to draw definite conclusions regarding the details of the core microbiome in the human tonsillar crypts. In a very comprehensive study of faecal samples from 154 individuals by Turnbaugh et al. [19], no single bacterial phylotype was detected in all samples. Yet, a core gut microbiome seems to exist at the level of metabolic functions, suggesting that microbial function rather than phylogeny is conserved across individuals.

It is striking that corynebacteria and coagulase-negative staphylococci, which are significant components of the microbiota of the upper respiratory tract in general, were found in only small proportions in the tonsillar crypts, in support of our expectations that the crypts constitute a unique habitat more reminiscent of the periodontal microbiota [41] than that of traditional mucosal surfaces. The study showed significant differences between the tonsillar crypt microbiota in healthy adults and adults with recurrent tonsillitis, and somewhat different community structures in children with recurrent tonsillitis and children with tonsillar hyperplasia. Although studies involving more subjects are required, these results support the hypothesis that recurrent tonsillitis may be caused by a shift in the microbiota of the tonsillar crypts but with significant individual differences.

Like in other infectious diseases associated with a complex microbiota, identification of a specific etiologic agent is difficult. One of the striking differences between healthy adults and adults with recurrent tonsillitis was the presence of *Fusobacterium necrophorum* in the latter. During the last decade, *F. necrophorum* has attracted attention due to a possible association with tonsillitis, particularly recurrent tonsillitis [9], [10], [42], and as the etiology of the severe Lemierre’s syndrome [43]. The syndrome begins with a pharyngotonsillitis and/or peritonsillar infection followed by unilateral swelling and tenderness along the sternocleidomastoid muscle owing to thrombophlebitis of the internal jugular vein. Within one week patients may develop severe postanginal *F. necrophorum* septicemia with rigors, high fever, and metastatic infections, especially in the lungs and bones [44]. Although, the syndrome is often considered rare, there has been an increase of reported cases over the last two decades, and the annual incidence in Denmark has been estimated to 3.6 cases per million. In young adults aged 15–24 years, the annual incidence may be as high as 14.4 cases per million [45], which is five times higher than the incidence in the same age-group of acute rheumatic fever caused by *S. pyogenes* [42]. The syndrome generally requires hospitalization for more than three weeks, and even today the mortality-rate is around 7% [44]. *F. necrophorum* was assumed not to be part of the normal microbiota in human tonsils [46], but recent studies using culture-based and culture independent techniques (qPCR) revealed the presence of *F. necrophorum* in throat swabs of healthy tonsils [10], [47]. Such results, particularly PCR-based results, may be biased and do not reveal relative proportions of the target species within a complex microbiota. The present study, which applied the most sensitive and objective culture-independent method available, showed that *F. necrophorum* was almost completely absent in the tonsillar crypts of children and healthy adults, but present in significant quantities in all samples from adults suffering from recurrent tonsillitis. This supports the hypothesis that *F. necrophorum* is etiologically involved in recurrent or chronic tonsillitis in young adults, and that tonsils constitute the primary focus of Lemierre’s syndrome. However, due to the complexity of the microbiota, a polymicrobial etiology of recurrent tonsillitis in adults cannot be excluded.

Previous culture-based studies of the bacteriology of recurrent tonsillitis focused mainly on bacteria that are recognized as etiological agents of acute tonsillitis, i.e. *S. pyogenes*, *S. aureus, S. pneumoniae,* and *H. influenzae*. In most cases of recurrent tonsillitis these species may be isolated from the tonsillar core [47]–[49]. We found substantial proportions of *S. pneumoniae* in two samples from children and *H. influenzae* in most children. However, sequences assigned to the genus *Staphylococcus* accounted for less than 0.1% of the total number of sequences and *S. pyogenes* and *Streptococcus dysgalactiae* subp. *equisimilis* only constituted minor proportions both in children and adults. While methodology, in particular the DNA extraction, primer design, and the differences in amplification and sequence affinity among different bacteria, to some extent affects the results obtained, sequence-based analyses are still more objective than previously used culture-based methods. Thus, our results suggest that culture-based studies may overestimate the abundance and clinical significance of *S. pyogenes, S. dysgalactiae* subp. *equisimilis* and *S. aureus* in children and adults with recurrent tonsillitis.

*H. influenzae* has been associated with recurrent tonsillitis [13] and hyperplastic tonsils in children [49]. We found that *H. influenzae* was present in major proportions in eight out of 10 samples from children (>2% of total sequences), with no differences in the abundance between tonsillar hyperplasia and recurrent tonsillitis. The same was true for *H. haemolyticus,* which, in contrast to *H. influenzae*, is considered of low pathogenic potential [50]. Our results suggest that the tonsillar crypt is the primary habitat of this species. In contrast, the species composition of *Neisseria* differed between samples from children with tonsillar hyperplasia and samples from children with recurrent tonsillitis. *N. lactamica,* found exclusively in children with tonsillar hyperplasia and in very low proportions in adults, was previously shown to ensure immunological “non-responsiveness” in the host due to expression of a B cell superantigen with high affinity for surface IgM and IgD. This allows *N. lactamica* to specifically activate CD5^+^ B1 cells, which produce natural antibodies that preclude activation of the adaptive immune system [51], [52].

*Spirochaetes,* particularly *Treponema,* have been linked to chronic tonsillitis in a Belgian study [53], and *T. denticola* is a member of the so-called “red-complex” of bacterial species that are strongly associated with periodontitis [36]. Interestingly, *Treponema*, mostly *T. medium* and *T. denticola* were almost exclusively detected in adults, and especially in healthy adults. *Porphyromonas gingivalis*, which also is part of the “red-complex” of subgingival bacteria associated with periodontal disease, was present only in one healthy adult. In contrast, *P. catoniae* was found in all samples and was together with the yet uncultured *Porphyromonas* genomospecies PAJ1 which, according to a BLAST search, previously was detected in such diverse habitats as the ileum, the distal esophagus, and the human skin, but also in saliva, subgigival plaque, and in lower respiratory tract samples from cystic fibrosis patients the only *Porphyromonas* species detected in children [54]–[56]. Interestingly, *P. catoniae* is an early colonizer of the periodontal crevices [57] and has been suggested as an oral health marker [58], [59], which is in agreement with its common presence in non-inflamed tonsils.

*Prevotella intermedia* has been previously associated with recurrent tonsillitis, supported by the demonstration of serum antibodies reactive with *P. intermedia* in patients with recurrent non-streptococcal tonsillitis [60]. In our study *P. intermedia was* virtually absent, except for one healthy adult (sample 13), where the species was found in very large proportions (84.6%). As a potential contributor to tonsillar inflammation, *P. melaninogenica*/*P. histicola* (indistinguishable by sequence analyses) were found in significantly larger quantities in samples from adults with recurrent tonsillitis that in healthy adults. This is in agreement with previous findings of larger proportions of *P. melaninogenica* in children with recurrently inflamed tonsils compared to normal tonsils [61]. *P. melaninogenica* is recognized as one of the β-lactamase producers [62] that may be found in large quantities in tonsils [63], which explains why penicillin may be inefficient for treatment of recurrent tonsillitis [64] as β-lactamase producing anaerobic bacteria may protect other pathogenic bacteria from being eradicated by penicillin.

In conclusion, our results demonstrated a core microbiome of relative few, proportionally significant genera in all tonsillar crypt samples regardless of age and health status, but with significant differences in relative proportions in individuals both during health and disease. Unifrac analysis showed that recurrent tonsillitis is associated with a shift in the bacterial composition in the tonsillar crypts. Using a novel approach for the assignment of sequences to species level, we were able to demonstrate hitherto unknown details in the microbiota, including significant differences between children and adults. *F. necrophorum, S. intermedius,* and *P. melaninogenica*/*P. histicola* were significantly associated with recurrent tonsillitis in adults while species traditionally associated with acute tonsillitis, i.e. *S. pyogenes, S. dysgalactiae* subsp. *equisimilis,* and *S. aureus,* were found in very low proportions. These detailed maps of the tonsillar crypt microbiota in health and disease provide an improved basis for understanding the complex host-parasite relationships in a distinct and important habitat of the human body with intimate connections to the immune system.

## Materials and Methods

### Subjects

A total of 20 samples from 20 patients admitted to the Ear-Nose-Throat Department at Gentofte Hospital, Denmark, were examined. Ten samples were from children aged 2 to 4 years, while 10 samples were from adults aged 18 to 35 years. Five samples each from children and adults were collected during tonsillectomy due to recurrent tonsillitis. The remaining five samples from children were collected during tonsillectomy due to tonsillar hyperplasia but without current or previous acute or recurrent tonsillitis. Five samples from healthy adults without a history of episodes of acute or recurrent tonsillitis served as healthy controls and were collected during surgery other than tonsillectomy. These five controls were treated for a vocal cord polyp (two patients), benign throat tumour (one patient), and ear (one patient) and septum plastic surgery (one patient) (see Table 1). Patients were excluded from the study if they had received antibiotics one month prior to the sample collection or were in treatment with immunosuppressive drugs. At the time of sampling, none of the included patients had an acute throat infection. Recurrent tonsillitis was defined as three or more occurrences of acute tonsillitis per year.

Samples from the tonsillar crypts were taken during anaesthesia, but before surgery with a sharp, small surgical spoon. Special attention was taken not to touch the outer surface of the tonsil with the spoon. Samples were collected from two crypts from the same tonsil and pooled in cryotubes containing 400 µl of ATL-buffer (Qiagen) immediately frozen at −20°C. Samples were then transported frozen on dry ice from Gentofte Hospital to Aarhus University where all analyses were performed.

### Ethics Statement

The study protocol was approved by The Danish Scientific Ethics Committee (Reference 20080224), and written informed consent was obtained from patients or from parents of the children included in the study.

### DNA Extraction

After thawing, 45 µl of a proteinase K solution (10 µg/µl) were added to the sample and incubated at 55°C for two hours. Next, the samples were transferred to a Lysing Matrix Tube E (MP Biomedicals) and 500 µl of TE-buffer was added. Extraction of DNA from the samples was done on a Fastprep FP120 (Thermo Savant) at 5.5 ms^−1^ for 30 seconds. The treatment was repeated three times and the samples were cooled on ice between treatments. Finally, the samples were centrifuged for 5 min at 3000×g and the supernatant was then transferred to three 1.5 ml tubes (Eppendorf) with 200 µl in each. The final extraction of DNA was made using the Qiagen Blood and Tissue kit and the Animal Tissue protocol from step 3 following the manufacturer’s instructions (Qiagen). The DNA was eluted in 100 AE buffer.

### PCR and Pyrosequencing

Partial 16S rRNA gene sequences were amplified from the samples using the barcoded-primer approach to multiplex pyrosequencing. Using the 530F-mod primer (5′-Fusion A adaptor-Barcode-GCCAGCMGCNGCGGTA-3) and the 1061R primer (5′-Fusion B adaptor-CRRCACGAGCTGACGAC-3) a 562 bp DNA fragment spanning the V4–V6 region of the 16S rRNA gene was amplified by PCR [22], which allow sequence analyses of the V4–V5 region. These primers were used with success in other microbial diversity studies using the pyrosequncing approach [22], [23]. In silico evaluations have predicted that the V3–V4 and V4–V5 regions would provide the highest classification accuracy for the pyrosequencing technology [24] as well as the lowest base error rate in 454-pyrosequencing compared to the V6/V9 region [25]. The fusion adaptors were for the GS FLX Titanium emPCR (Lib-L) Kit and the 10-bp barcodes were the Multiplex Identifier (MID), MID1 to MID8, from Roche. PCR reaction mixtures were carried out in a total volume of 25 µl and comprised of 10 µl of diluted DNA sample, 2.5 µl 10× PfuUltra II reaction buffer (Stratagene), 400 nM (each) of each primers (IDT), 0.5 µl PfuUltra II fusion HS DNA polymerase polymerase (Stratagene), 1 µl dNTP mix (25 mM each dNTP), and 9 µl of molecular-biology grade water. PCR was performed using the following cycle conditions: an initial denaturation at 95°C for 2 minutes, followed by 30 cycles of denaturation at 95°C for 20 sec, annealing at 55°C for 20 s, elongation at 72°C for 15 s, and then a final elongation step at 72°C for 3 min. Six PCR reactions were performed for all samples. The PCR products from each of the six reactions from each sample were verified on an agarose gel. The correct band was then gel excised, pooled and purified using the NucleoSpin® Extract Kit (Macherey-Nagel). The concentration of the purified PCR product was measured by a NanoDrop 2000 spectrophotometer (Thermo Scientific). The 16S rRNA amplicons were sequenced unidirectionally from the forward primer end using titanium chemistry and two two-region GS FLX Standard Pico TiterPlates (70X75) on a 454 Genome Sequencer FLX platform at 454 Life Sciences (Branford, CT, USA). All sequences were deposited to the NCBI Sequence Read Archive (SRA) with the accession number SRA053229.

### Sequence Processing and Statistical Analysis

The open-source, platform-independent, community-supported software program, mothur v.1.22.2 (http.//www.mothur.org) was used to process and analyse the pyrosequencing data [26]. Processing of raw pyrosequencing data was done according to the Schloss standard operating procedure (SOP) (http://www.mothur.org/wiki/Schloss_SOP) [25]. Briefly, sequencing noise was initially reduced using the shhh.flows command, which is an implementation of the AmpliconNoise algorithm into the mothur software package [27]. Sequences with more than two mismatches to the primer sequence, one mismatch to the barcode sequence, containing more than eight homopolymers, or containing any ambiguous characters were removed before denoising of the sequences. The maximum number of flows was set to 720 as recommended by Quince et al. [27]. After denoising, sequences were aligned to the Silva reference alignment and the resulting alignment was then filtered so that all of our sequences only overlapped in the same region. Furthermore, sequences shorter than 350 bases were removed. To further reduce sequencing errors a preclustering step implementing a pseudo-single linkage algorithm originally developed by Huse et al. [28] was performed. Chimers were removed using the build-in version of the UCHIME algorithm in mothur [29]. Lastly, singletons and duplicates were removed before analysis to preclude inclusion of sequences from potential contamination or sequencing errors. All samples were rarefied to a sequencing depth of 4599 reads per sample prior to downstream analyses using the Sub.sample command in mothur. However, all sequences that passed the quality control in each sample were used for the phylogenetic analysis of sequences to species level.

Preliminary sorting of the sequences was done by clustering the sequences into operational taxonomic units (OTU) defined by a 3% distance level using the average neighbor clustering algorithm. Invsimpson and the Shannon diversity indexes were calculated in mothur. Weighted UniFrac analyses were done by generating a phylogenetic tree using the implementation of the Clearcut program (http://bioinformatics.hungry.com/clearcut/) into the mothur software.

Classification of the sequences into phyla and genera was done using the Bayesian method and the taxonomic outline from RDP. The confidence cut-off was set to 80%. To assign the sequences to species we performed phylogenetic analysis of unique 16S rRNA sequences with inclusion of 16S rRNA gene sequences of relevant designated type strains. The phylogenetic tree construction and bootstrap analyses using 500 replicates were conducted in Mega v. 5.05 [30]. Sequences that formed coherent and distinct clusters together with a single type strain were assigned to the corresponding species. If diffuse clusters included several type strains, we assigned the sequences to specific taxa or groups of taxa, whenever possible, by detecting species-specific sequences signatures identified according to recently described principles [31]. The signatures were initially identified based on alignments of 16S rRNA sequences of type and reference strains and validated by BLAST analysis in the Ribosomal RNA Database Project version 10 and the NCBI nucleotide sequence database. Sequence signatures used to assign sequences to species level are shown in Table S1. Sequences for the phylogenetic analysis were obtained from the total number of sequences by the “*get.lineage*” command in mothur, which allows extraction of sequences classified to the same taxa, e.g. family or genus by the “*classify.seq*s” command in mothur. Sequences not classified to genus level by the automatic classification were included in the phylogenetic analysis where appropriate. Based on abundance and importance, sequences from the following taxa were identified to species level by this approach: *Streptococcaceae, Neisseriaceae, Fusobacterium, Pasteurellaceae, Prevotella, Porphyromonadaceae, Treponema, Flavobacteriaceae, Veillonellaceae, Gemella, Rothia, Parvimonas, Granulicatella, Tannerella,* and *Actinomyces.* Also included in the phylogenetic analyses were sequences that were not assigned to genus level by the automatic classification in Mothur. These included sequences assigned as unclassified *Neisseriaceae,* unclassified *Pasteurellaceae,* unclassified *Flavobacteriaceae,* and unclassified *Bacteroidales.* Sequences assigned to genomospecies by the phylogenetic analysis were also blasted against the Human Oral Microbiome Database (HOMD) (www.HOMD.org) [32]. GenBank accession numbers for the sequences of the genomospecies are found in Table S3.

Metastats was used to detect differentially abundant features between children and adults, children with hyperplasia and children with recurrent tonsillitis, and healthy adults and adults with recurrent tonsillitis [33]. Taxa were considered differentially abundant when p<0.05 and q<0.05. Student’s t-test was used to detect differences in invsimpson and Shannon diversity indexes between the groups. Differences were considered significant at p<0.05. Molecular variance (AMOVA) was applied to test if separation of the defined groups visualized by the Principal Coordinates (PCoA) plot was statistically significant. Differences were considered significant at p<0.05.

## Supporting Information

Table S1 **Sequence signatures in 16S rRNA genes used for identification of species and groups of species of** ***Streptococcus*** **,** ***Prevotella*** **,** ***Haemophilus*** **,** ***Aggregatibacter, Actinomyces,*** **and** ***Veillonellaceae*** **combined with phylogenetic analyses.**
(DOCX)

Table S2 **Percentage proportions of phyla, genera and species in each sample.** The identified species constituted between 70% and 99% (mean: 90%) of the total number of sequences in each sample. Significant differences calculated using Metastats between groups are highlighted in **bold.** See Material and method for the description of the methods used for species identification of the sequences.
(XLSX)

Table S3 **Representative sequences of the uncultured genomospecies blasted against the Human Oral Microbiome Database (HOMD) and GenBank.** GenBank accession numbers of representative sequences are listed.
(XLSX)
