# Supplementary material for: Molecular Mapping to Species Level of the Tonsillar Crypt Microbiota Associated with Health and Recurrent Tonsillitis
Source: PLoS One. 2013 Feb 21;8(2):e56418. doi: 10.1371/journal.pone.0056418 (PMC3578847; doi:10.1371/journal.pone.0056418)
Supplement: Table S1 — Sequence signatures in 16S rRNA genes used for identification of species and groups of species of Streptococcus , Prevotella , Haemophilus , Aggregatibacter, Actinomyces, and Veillonellaceae combined with phylogenetic analyses. (DOCX) [file pone.0056418.s001.docx]

| **Species** | **Signature** |
| --- | --- |
| *Streptococcus pneumoniae* | GTAGTCCACGCTGTAAACGATGAGT |
| *Streptococcus pseudopneumoniae* | GACCCTTTCCGGGGTTTAGTGCCGT excluding GTAGTCCACGCTGTAAACGATGAGT |
| *Streptococcus mitis/S. oralis/S. infantis* | TGTGGCTTAACCATAGTACGCTTTGGAAACTGTTTAACTTGAGTGCAAGAGGGGAGAGTGGAATT |
| *Streptococcus anginosus/S. constellatus/S. intermedius* | TTGTAGGCTTTGGAAACTGTTTAACTTGAGTGCAGA |
| *Streptococcus agalactiae* | TTAACCATTGTACGCTTTGGAAACTGG |
| *Streptococcus dysgalactiae* *subsp. equisimilis* | TCAACCATTGTACGCTTTGGAAACTGG |
| *Lactococcus lactis* | GCAGGTGGTTTATTAA |
| *Prevotella melaninogenica / P. histicola* | TAGGCTGGAGATTAAGT |
| *Prevotella fusca/ P. denticola / P. multiformis* | TAGGCCGGGGATTAAGT |
| *Prevotella veroralis* | TTCGGGCGTTATCCGGATTTATTGGGTTTAAAGGGAGCGTAGGCCGGAGATTAAGT |
| *Prevotella scopus* | TCCAGGCGTTATCCGGATTTATTGGGTTTAAAGGGAGCGTAGGCCGGAGATTAAGT |
| *Haemophilus haemolyticus* | AAGCTTGGTGCCCGT |
| *Haemophilus influenzae* | TTTCAGACTGGGTAACT excluding AAGCTTGGTGCCCGT |
| *Haemophilus parainfluenzae* | CGATGTCGATTTGGGGGTTGA |
| *Haemophilus parahaemolyticus* | CGATGTCGATTTGGGGA |
| *Haemophilus pittmaniae* | GGACTTTTAAGTGAGGTGTGAAAG |
| *Aggregatibacter aphrophilus* | GGACTTTTAAGTGAGGTGTGAAAT |
| Aggregatibacter segnis | TCCCCGGGCTTAACCTGGGAATTGCATTTCAGACTGGGTAGC |
| *Neisseria polysaccharea* | ACTGCGTTCTGAACTGGGTGACTCGAGTGTGTCAGAGGGAGGTAG |
| *Eikenella corrodens* | AACCTGGGAACTGCGTTCTGAACTGGAT |
| *Actinomyces odontolyticus* | GTGCGGTGGGTACGGGCT/AGA/GCTTGAGTGCGGTAGGGGAGACTGGAAC |
| *Actinomyces massiliensis* | CTCAGCTGGGGGCGTGCGGTGGGTACGGGCTGGCTTGAGTGCGGTAGGGGAGA |
| *Actinomyces israelii/A. gerencseriae* | GGTAGTCCATGCTGTAAACGTTGGGCACTAGGTGTGGGGGGTC |
| *Anaeroglobus_geminatus* | TGAGAGGATGGAAACTGGACAGCTGAGAGTGTCGGA |
| *Megasphaera micronuciformis* | AACTGGGAAGCTCAGAGTATCGGAGAGGAAAGCGG |
| *Selenomonas sputigena* | CCTGGCCGTAAACGATGAATGCTAGGTGT |
| *Selemonas infelix* | CGTGATGGGAAAGAAACTACATGTCTTGAGTAC + TTGGGAGGAACACCAGT |
| *Dialister invisus* | CTTCCCAAGTCCCTCTTAAAAGTGCGGGGCTTAA |
| *Dialister pneumosintes* | AAGCGTGGGGCTCAACCCCATGAGGGGATGGAAACT |
| *Centipeda periodontii* | CGTGATGGGAAAGAAACTACATGTCTTGAGTAC + TGGGAGGAACACCAGTGG |

**Table S1.** Sequence signatures in 16S rRNA genes used for identification of species and groups of species of *Streptococcus*, *Prevotella*, *Haemophilus*, *Aggregatibacter, Actinomyces,* and *Veillonellaceae* combined with phylogenetic analyses.
